# Use of Supervision Data to Improve Quality of Care for Malaria in Pregnancy: Experience in Six African Countries

**DOI:** 10.4269/ajtmh.23-0206

**Published:** 2023-12-26

**Authors:** Katherine Wolf, Jadmin Mostel, Lolade Oseni, Patricia Gomez, Tabitha Kibuka, Courtney Emerson, Julie R. Gutman, Ashley Malpass, Susan Youll, Jean Yves Mukamba, Eric Tchinda Meli, Dorothy Achu, Paul Tjek, Jean Louis Assa, Mamadou Silue, Méa Antoine Tanoh, Colette Kokrasset-Yah, Felicia Babanawo, Amos Asiedu, Mildred Komey, Paul Boateng, Maureen Mabiria, Augustine Ngindu, Peter Njiru, Ahmeddin Hassan Omar, Fatoumata A. Sidibe, Chebou Diallo, Beh Kamate, Aissata Kone, Sanoussi Elisha, Arouna Djibrilla Maiga, Alzouma Ibrahim Mayaki, Fati Tidjani Issa Gana, Gladys Tetteh

**Affiliations:** ^1^ PMI Impact Malaria Project, Jhpiego, Baltimore, Maryland;; ^2^ PMI Impact Malaria Project, Population Services International, Washington, District of Columbia;; ^3^ U.S. President’s Malaria Initiative, Malaria Branch, Centers for Disease Control and Prevention, Atlanta, Georgia;; ^4^ U.S. President’s Malaria Initiative, United States Agency for International Development, Washington, District of Columbia;; ^5^ PMI Impact Malaria/Cameroon, Yaoundé, Cameroon;; ^6^ Ministry of Health, Yaoundé, Cameroon;; ^7^ PMI Impact Malaria/Cote d’Ivoire, Abidjan, Cote d’Ivoire;; ^8^ Ministry of Health, Abidjan, Cote d’Ivoire;; ^9^ PMI Impact Malaria/Ghana, Accra, Ghana;; ^10^ Ministry of Health, Accra, Ghana;; ^11^ PMI Impact Malaria/Kenya, Nairobi, Kenya;; ^12^ Ministry of Health, Nairobi, Kenya;; ^13^ PMI Impact Malaria/Mali, Bamako, Mali;; ^14^ Ministry of Health, Bamako, Mali;; ^15^ PMI Impact Malaria/Niger, Niamey, Niger;; ^16^ Ministry of Health, Niamey, Niger

## Abstract

Malaria in pregnancy (MiP) intervention coverage, especially intermittent preventive treatment in pregnancy (IPTp), lags behind other global malaria indicators. In 2020, across Africa, only 32% of eligible pregnant women received at least three IPTp doses, despite high antenatal care attendance. We conducted a secondary analysis of data collected during Outreach Training and Supportive Supervision visits from 2019 to 2020 to assess quality of care and explore factors contributing to providers’ competence in providing IPTp, insecticide-treated nets, malaria case management, and respectful maternity care. Data were collected during observations of provider–patient interactions in six countries (Cameroon, Cote d’Ivoire, Ghana, Kenya, Mali, and Niger). Competency scores (i.e., composite scores of supervisory checklist observations) were calculated across three domains: MiP prevention, MiP treatment, and respectful maternity care. Scores are used to understand drivers of competency, rather than to assess individual health worker performance. Country-specific multilinear regressions were used to assess how competency score was influenced by commodity availability, training, provider gender and cadre, job aid availability, and facility type. Average competency scores varied across countries: prevention (44–90%), treatment (78–90%), and respectful maternity care (53–93%). The relative association of each factor with competency score varied. Commodity availability, training, and access to job aids correlated positively with competency in multiple countries. To improve MiP service quality, equitable access to training opportunities for different cadres, targeted training, and access to job aids and guidelines should be available for providers. Collection and analysis of routine supervision data can support tailored actions to improve quality MiP services.

## INTRODUCTION

Malaria in pregnancy (MiP) is a serious public health threat that can lead to preterm deliveries, low birthweight, and neonatal death.^1^ To decrease the risks of MiP, the WHO recommends that pregnant women in areas of moderate to high malaria transmission receive at least three doses of intermittent preventive treatment of malaria in pregnancy (IPTp) using sulfadoxine–pyrimethamine (SP), use insecticide-treated nets (ITNs), and receive prompt diagnosis and treatment of MiP.^2^^,^^3^ Although there have been improvements in the uptake of these interventions, in 2020 only 57% of eligible pregnant women in 33 countries in Africa received at least one dose of IPTp and only 32% received three doses.^4^

Many of the interventions aimed at reducing MiP are focused on the behaviors of pregnant women, such as promoting early and regular antenatal care (ANC) attendance and sleeping under an ITN.^5^ However, the quality of MiP prevention and treatment care depends primarily on provider knowledge and behaviors, and onsite resources and commodities. Providers are responsible for distributing ITNs to women during their first ANC visit, supplying ongoing counseling on consistent ITN use, and ensuring that women receive IPTp doses as early as possible during their second trimester and at every subsequent ANC visit, at least 4 weeks apart. Providers at antenatal and curative service departments are also responsible for prompt testing and treatment of MiP. Although many of these interventions require pregnant women to seek care, the actual provision is dependent on the actions of the provider and the availability of commodities, as well as data capture and reporting, all of which contribute to the measurable overall quality of care (QoC).^6^

Quality of care is defined by the WHO as “the extent to which health care services provided to individuals and patient populations improve desired health outcomes. In order to achieve this, health care must be safe, effective, timely, efficient, equitable and people-centered.” (p.14)^7^ High-quality care ensures patients receive all the interventions and information needed to benefit from health services. Previous studies have documented the overall poor QoC for malaria^8^ and the need to overcome health system barriers.^9^ Quality of care for pregnant women is often measured by the number of ANC contacts, the timing of the first ANC visit, and the provision of all recommended ANC services.^10^ However, there are additional components of QoC that can contribute to or strengthen the overall QoC, including those relevant to this analysis: respect and preservation of dignity, competent and motivated human resources, and essential physical resources available.^7^ Previous research on the MiP QoC topic has found that training of providers alone or in conjunction with supportive supervision, particularly training that incorporates clinical practice, can result in improvements in health worker performance^11^ and competency in delivering MiP services.^12^ Ensuring commodity availability is also critical to delivering appropriate care.^13^ In addition, respectful maternal care (RMC), a framework for the interpersonal care that women receive during their pregnancy, has the potential to improve health outcomes^14^ and is being incorporated increasingly into QoC measures.^15^ Respectful care encompasses respect for women’s basic human rights, including respect for women’s autonomy, and preservation of respect, dignity, feelings, choices, and preferences.^7^ When women have positive perceptions of the quality of the care they receive, they are more likely to return for follow-up appointments.^16^ In turn, women who were often treated in a humane, respectful, supportive environment are recorded as having the highest percentages of adequate ANC attendance.^17^

The U.S. President’s Malaria Initiative (PMI) Impact Malaria Project works across 19 countries to improve malaria service delivery by supporting national malaria control and elimination programs to engage with a four-prong quality improvement approach. The cornerstone of this strategy is Outreach Training and Supportive Supervision Plus (OTSS+), a facility-level approach aimed at improving health facility and provider competency through onsite supportive supervision, coaching, troubleshooting, and on-the-job training. This approach has been found to be an effective performance appraisal system that can improve the quality of services.^18^^,^^19^ Providers are deemed “competent” if they attain a score ≥90% on one of the standard checklists used to assess health facility readiness or health worker performance.

Our analysis did not use a competency threshold; the research questions did not seek to determine whether competency was achieved, but rather to assess elements that influence competency scores. To assist countries in identifying and addressing gaps and causes of missed opportunities in MiP service delivery, MiP-focused OTSS+ data from six countries (Cameroon, Cote d’Ivoire, Ghana, Kenya, Mali, and Niger), collected between 2019 and 2020, were analyzed to assess provider competency and QoC for MiP prevention and treatment, and for RMC. These countries were selected based on the availability of OTSS+ data at the time of analysis through the PMI Impact Malaria Project. Each country conducted OTSS+ in different levels of facilities based on supportive supervision strategies made at the national level, and included health centers and hospitals in Cameroon; dispensaries, health centers, and hospitals in Cote d’Ivoire; dispensaries, health centers, and hospitals in Ghana; dispensaries, health centers, and hospitals in Kenya; and health centers in Mali and Niger.

## MATERIALS AND METHODS

### Data collection.

A secondary analysis of data collected between 2019 and 2020 during rounds of OTSS+ (Supplemental Table S1) was conducted to assess QoC and explore the factors contributing to health workers’ competency in prevention of MiP, treatment of MiP, and RMC. The OTSS+ data were collected by teams in health facilities supported by Impact Malaria, comprised of ministry of health representatives, who performed observations of provider–patient interactions using a package of checklists to monitor health worker performance and health facility readiness for malaria service delivery, with support from Impact Malaria Project staff. To facilitate real-time access, this assessment used electronically collected data, stored on cloud-based platforms, from one health facility readiness checklist and one MiP checklist. These checklists were based on a standard template; each country edited the checklists to fit their national context. Although not all checklist questions included in our analysis are worded identically across countries, they are comparable in nature. Where differences exist, this is noted in the presentation of data. Not all the same health facilities were observed during each OTSS+ round.

### Data cleaning and variable selection.

The first step of the MiP QoC data-cleaning process identified the data elements from the MiP and facility readiness OTSS+ checklists to use for the analysis. Prevention competency in MiP included supervision checklist observations of greeting and respectful care, patient history taken, IPTp administration, ITN distribution, and other ANC interventions. Thirty-two binary checklist questions were averaged to generate a competency score. Treatment competency in MiP included supervision checklist observations of greetings, medical history taken, diagnosis, and correct treatment. Twenty-five binary checklist questions were averaged to generate a competency score. Competency in RMC included supervision checklist observations of the woman being greeted, invited to sit down, treated with respect, and asked whether she had questions. The facility readiness module provided information on stock status, provider training, and the availability of guidelines and job aids. Elements deemed most representative of influencing competency scores of health workers and providing the broadest scope were selected to answer the research questions. Data were de-identified, and select data were imported into spreadsheets. Data availability was confirmed, and data element selection was refined further to respond to research questions of interest. All personal identifiable information was masked, and data from the two OTSS+ checklists were merged based on facility name (complete match) and date of observation (approximate match) in Stata 15.1 (StataCorp, College Station, TX) (Supplemental Table S2).

### Data analysis.

#### Descriptive statistics.

The unit of analysis was supervision observations derived from the checklists of interest. Descriptive statistics were calculated for a variety of data elements to assess proportions of these elements. Calculations of the descriptive statistics, and for the statistical analysis, were performed using data sets aggregated by country (e.g., data analyses were not disaggregated by OTSS+ round) (Supplemental Figure S1).

#### Regression analyses.

Multilinear regressions were performed to assess how the MiP prevention competency score (Supplemental Table S3), MiP treatment competency score (Supplemental Table S4), and RMC score (Supplemental Table S5) were influenced by the independent variables of interest, including commodity (SP, ITNs, rapid diagnostic tests, quinine, artemisinin-based combination therapy [ACT], and injectable artesunate) availability, training, gender, job aid availability, cadre of health worker, and facility type (see definitions in Supplemental Tables S6 and S7). To control for any confounding effects of commodity availability, multivariate analyses were conducted when assessing the relationships of noncommodity variables with MiP prevention, MiP treatment, and RMC competencies. This was done by including the ACT availability variable for MiP treatment regressions and including the SP availability variable for MiP prevention regressions. When assessing the relationship between commodity availability and MiP prevention and MiP treatment competencies, confounding effects from other commodities were also controlled by using multivariate analyses. Two predictor variables, the availability of SP and the availability of ITNs, were regressed in a multivariate model against the dependent variable of the MiP prevention competency score. Three predictor variables—the availability of quinine tablets, injectable artesunate, and ACTs—were regressed in a multivariate model against the dependent variable of the MiP treatment competency score. Prevention and treatment competency scores for MiP for each observation were existing variables in the countries’ OTSS+ data sets and were autogenerated based on observation of health workers providing the critical elements of prevention and treatment included in the OTSS+ checklist. Of note, the analyses for Ghana did not include any models with the MiP treatment score because the OTSS+ model in that country does not collect MiP treatment data. The RMC score was calculated manually by averaging available binary data elements for each observation on whether the patient was greeted, invited to sit down, treated with respect, and asked whether she had questions (Supplemental Table S3).

### Data validation.

Country data validation meetings were held after the completion of data analysis and yielded qualitative information about data collection, the health system, and the supervision process to contextualize country-specific findings of the quantitative data analysis. Each country’s validation meeting was held virtually and included the study team and Impact Malaria teams in each country office. Impact Malaria country teams were requested to share the results with relevant counterparts in the ministries of health’s malaria and maternal health departments for additional feedback. Qualitative data from these discussions were applied to understand quantitative findings more fully.

## RESULTS

Our analysis was performed using data from six countries and a total of 2,444 facility observations: 339 from Cameroon, 284 from Cote d’Ivoire, 951 from Ghana, 82 from Kenya, 595 from Mali, and 193 from Niger. The number of significant correlations observed was related directly to the number of observations in the country’s data set.

### Health worker competency.

**Figure 1.**
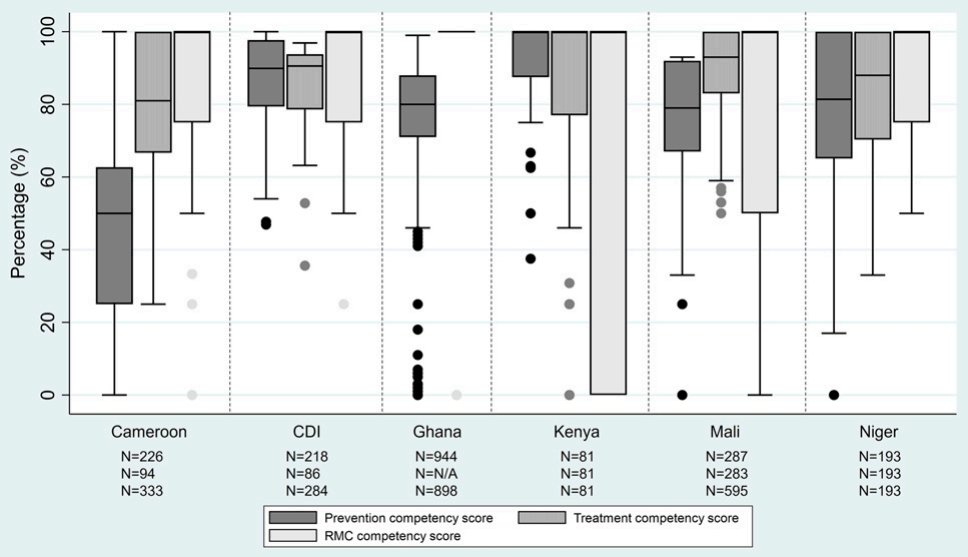
Distribution of malaria in pregnancy (MiP) prevention competency, MiP treatment competency, and respectful maternal care (RMC) competency scores. From bottom to top of each range: minimum value, first quartile value, median value, third quartile value, and maximum value. CDI = Cote d’Ivoire.

Average health worker competencies differed by country, from 44% to 90% in MiP prevention, 78% to 90% in MiP treatment, and 53% to 93% in RMC (Figure 1).

### Commodity availability and competence.

Availability of SP and ITNs was high across countries, as was the availability of ACTs (except in Cameroon), whereas the availability of quinine and injectable artesunate varied considerably (Table 1).

**Table 1:** Availability of commodities for malaria in pregnancy prevention and treatment

Commodity	Cameroon, *n* (%)	CDI, *n* (%)	Ghana, *n* (%)	Kenya, *n* (%)	Mali, *n* (%)	Niger, *n* (%)
MiP prevention						
Sulfadoxine–pyrimethamine	319 (79)	278 (95)	934 (90)	82 (85)	595 (73)	187 (97)
Insecticide-treated nets	322 (71)	279 (90)	921 (70)	82 (79)	595 (77)	155 (85)
MiP treatment						
Quinine tablets	331 (68)	279 (20)	624 (36)	82 (11)	595 (89)	188 (73)
Injectable artesunate	330 (68)	279 (61)	569 (57)	82 (77)	595 (94)	188 (79)
Artemisinin-based combination therapy	332 (55)	279 (91)	871 (82)	82 (90)	595 (86)	188 (68)

CDI = Cote d’Ivoire; MiP = malaria in pregnancy.

Provider competency score in MiP service delivery was associated positively with the availability of MiP commodities, as demonstrated in the regression analysis; this relationship was statistically significant in several countries (Table 2). When SP was in stock, the MiP prevention competency score was 8% points greater in Cote d’Ivoire and 32% points greater in Ghana, compared with when SP was not in stock and when ITN stock availability was held constant. When both SP and directly observed therapy (DOT) supplies, consisting of water dispensers and cups, were in stock, MiP prevention competency was 8% points greater in Cote d’Ivoire and 31% points greater in Ghana. When ITNs were in stock, the MiP prevention competency score was 13% points greater in Cote d’Ivoire and approximately 17% points greater in both Ghana and Niger compared with when ITNs were not in stock and when SP stock availability was held constant. Notably, in Cameroon, the MiP prevention competency score was 11% points less when ITNs were in stock and when SP stock was held constant.

**Table 2:** A multivariate analysis of commodity availability with health worker competency score (as a percentage-point change) in delivery of malaria in pregnancy prevention and malaria in pregnancy treatment

Variable	Cameroon	CDI	Ghana	Kenya	Mali	Niger
MiP prevention						
SP*	−3.673^†^	7.977^‡^	31.999^§^	6.098^†^	1.819^†^	−12.195^†^
ITNs*	−11.333^ǁ^	13.300^§^	16.46^§^	0.758^†^	1.359^†^	16.594^ǁ^
SP and DOT supplies	2.811^†^	7.960^ǁ^	31.31^§^	5.433^†^	1.771^†^	0.899^†^
MiP treatment						
Quinine^¶^	0.595^†^	−5.243^†^	NA^#^	−16.60^†^	6.148^‡^	−0.050^†^
ACT/AL^¶^	2.822^†^	−1.592^†^	NA^#^	−6.856^†^	1.766^†^	8.666^ǁ^
Injectable artesunate^¶^	−2.172^†^	1.880^†^	NA^#^	4.483^†^	−0.603^†^	1.725^†^

ACT = artemisinin-based combination therapy; AL= artemether lumefantrine; CDI = Cote d’Ivoire; DOT = directly observed therapy; ITN = insecticide-treated net; MiP = malaria in pregnancy; NA = not applicable; SP = sulfadoxine-pyrimethamine.

* The dependent variable for this statistical regression is the MIP prevention competency score (with other variable effects held constant).

^†^ Not significant.

^‡^ *P <*0.05.

^§^ *P <*0.001.

^ǁ^ *P <*0.01.

^¶^ The dependent variable for this statistical regression is the MiP treatment competency score (with other variable effects held constant).

^#^ Ghana does not have treatment competency score data.

When quinine was in stock, the MiP treatment competency score was 6% points greater in Mali compared with when quinine was not in stock and when ACT and injectable artesunate stock availability were held constant. When ACTs were in stock, the MiP treatment competency score was 9% points greater in Niger compared with when ACTs were not in stock and quinine and injectable artesunate stock was held constant. Ghana was the only country included that reported clindamycin stock, which was present during 47% of observations.

### Provider and facility readiness and competency.

Among observed health worker cadres reported in Cameroon, Cote d’Ivoire, and Niger, 35% were nurses, 12% were nurse assistants (a cadre present/recorded only in Cameroon), 51% were midwives, and 2% each were doctors or other (Ghana, Kenya, and Mali did not collect data on provider cadre). Availability of MiP guidelines and job aids ranged from 49% to 88%, although only the Cote d’Ivoire checklist asked about both guidelines and jobs aids. The percentage of health workers who had been trained recently ranged from 27% in Cameroon to 85% in Cote d’Ivoire. Checklist question wording with regard to training status differed greatly between countries, based on national-level stakeholder checklist validation decisions.

**Figure 2.**
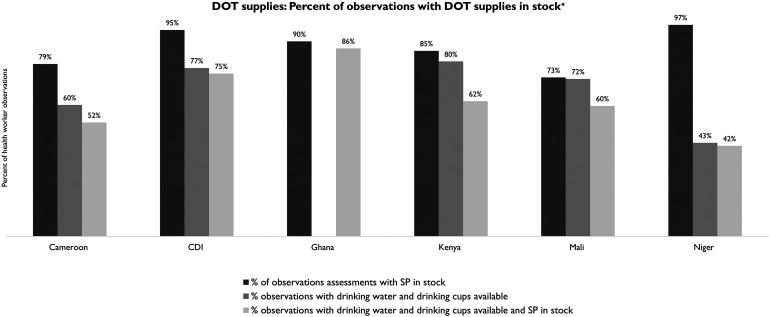
Directly observed therapy (DOT) supplies in stock on day of observation. *Ghana only asks: Is clean water available for DOT of sulfadoxine–pyrimethamine (SP) at the antenatal care unit? CDI = Cote d’Ivoire.

Although SP was available on the day of observation in most facilities across the six countries, many countries did not have adequate supplies of drinking water and cups to facilitate IPTp via DOT at ANC (Figure 2). Health facilities having the availability of all three resources on the day of observation (SP, drinking water, and cups at ANC) varied from 42% in Niger to 86% in Ghana. Of note, Ghana’s DOT supply data element only consisted of drinking water available for DOT. Ghana did not collect data on the availability of drinking cups.

### Malaria in pregnancy prevention.

A positive correlation was observed between DOT supplies (Cote d’Ivoire, Ghana), training (Cote d’Ivoire, Ghana), facility type (Cote d’Ivoire, Ghana), health worker gender (Cote d’Ivoire), and job aids (Cote d’Ivoire, Ghana) on health worker competency in the delivery of MiP prevention services (Table 3). Regression analysis also found a significant relationship between provider cadre and MiP prevention competency in Cote d’Ivoire, where midwives had lower competency scores than nurses. In Cameroon, both midwives and nurse assistants had greater competency scores than nurses.

**Table 3:** A multivariate analysis of noncommodity variables with health worker competency score (as a percentage-point change) in the delivery of malaria in pregnancy prevention

Variable	Cameroon	CDI	Ghana	Kenya	Mali	Niger
Recent training*	2.967^†^	7.699^‡^	11.927^‡^	NA^§^	3.253^†^	NA^§^
Job aids*	−3.122^†^	6.162^ǁ^	6.008^‡^	5.482^†^	−0.857^†^	7.971^†^
Provider gender*	0.351^†^	−5.657^ǁ^	NA^¶^	NA^§^	NA^§^	NA^§^
DOT supplies*	9.209^†^	5.329^#^	31.908^‡^	4.037^†^	−0.052^†^	0.843^†^
Cadre*						
Nurses	Ref.	Ref.	NA^§^	NA^§^	NA^§^	Ref.
Midwives	29.379^‡^	−5.740^ǁ^	NA^§^	NA^§^	NA^§^	0.107^†^
Nurse assistants	34.013^‡^	NA^§^	NA^§^	NA^§^	NA^§^	NA^§^
Facility type*						
Dispensaries	NA**	Ref.	Ref.	Ref.	NA^¶^	NA^¶^
Health centers	NA**	−7.078^ǁ^	7.710^‡^	6.481^†^	NA^¶^	NA^¶^
Hospitals	NA**	−8.618^†^	8.238^‡^	0.310^†^	NA^¶^	NA^¶^

CDI = Cote d’Ivoire; DOT = directly observed therapy; NA = not applicable; Ref. = reference value.

* The effect of sulfadoxine–pyrimethamine stock availability is held constant.

^†^ Not significant.

^‡^ *P <*0.001.

^§^ No available data.

^ǁ^ *P <*0.01.

^¶^ Mali and Niger only had observations at the health center facility level.

^#^ *P <*0.05.

** Observation sample sizes were widely disparate in Cameroon (health centers, *n =* 328; hospitals, *n =* 11).

### Malaria in pregnancy treatment.

For MiP treatment analyses, cadre and facility type data were not available for all countries. Ghana was not included in the MiP treatment analysis because they did not collect treatment competency score data in their OTSS+ checklist. There was a positive effect on MiP treatment competency of training and job aids in Cote d’Ivoire (8% and 9% points, respectively). No effect was observed for cadre, facility type, or provider gender on MiP treatment competency in any country (Table 4).

**Table 4:** A multivariate analysis of noncommodity variables with health worker competency score (as a percentage-point change) in the delivery of malaria in pregnancy treatment

Variable	Cameroon	CDI	Ghana	Kenya	Mali	Niger
Recent training*	−3.790^†^	8.332^‡^	NA^§^	NA^ǁ^	1.279^†^	NA^ǁ^
Job aids*	5.982^†^	8.882^¶^	NA^§^	10.75^†^	0.141^†^	8.729^†^
Provider gender*	4.284^†^	4.190^†^	NA^§^	NA^ǁ^	NA^ǁ^	3.161^†^
Cadre*						
Nurses	Ref.	Ref.	NA^§^	NA^ǁ^	NA^ǁ^	Ref.
Midwives	−10.284^†^	1.101^†^	NA^§^	NA^ǁ^	NA^ǁ^	1.519^†^
Nurse assistants	1.238^†^	NA^ǁ^	NA^§^	NA^ǁ^	NA^ǁ^	NA^ǁ^
Facility type*						
Dispensaries	NA^#^	Ref.	NA^§^	Ref.	NA**	NA**
Health centers	NA^#^	−5.581^†^	NA^§^	1.517^†^	NA**	NA**
Hospitals	NA^#^	3.176^†^	NA^§^	−2.662^†^	NA**	NA**

CDI = Cote d’Ivoire; NA = not applicable; Ref. = reference value.

* The effect of artemisinin-based combination therapy stock availability is held constant.

^†^ Not significant

^‡^ *P <*0.05.

^§^ Ghana does not have treatment competency score data.

^ǁ^ No available data.

^¶^ *P <*0.01.

^#^ Observation sample sizes were widely disparate in Cameroon (health centers, *n =* 328; hospitals, *n =* 11).

** Mali and Niger only have observations at the health center facility level.

### Respectful maternity care.

A significant effect on provider competency in delivery of RMC with regard to training (Ghana, Mali), facility type (Ghana), and MiP job aids (Cote d’Ivoire, Ghana) was observed. Cadre and gender were not observed to have a significant relationship with RMC competency scores in countries for which data were available (Table 5).

**Table 5:** A multivariate analysis of noncommodity influencers on health worker competency score (as a percentage-point change) in delivery of respectful maternal care

Influencer	Cameroon	CDI	Ghana*	Kenya*	Mali	Niger
Recent training^†^	−5.767^‡^	3.828^‡^	8.518^§^	NA^ǁ^	5.739^¶^	NA^ǁ^
Job aids^†^	−1.120^‡^	4.871^#^	4.117^#^	−7.488^‡^	0.305^‡^	2.911^‡^
Provider gender^†^	−0.104^‡^	−2.174^‡^	NA^ǁ^	NA^ǁ^	NA^ǁ^	3.724^‡^
Cadre^†^						
Nurses	Ref.	Ref.	NA^ǁ^	NA^ǁ^	NA^ǁ^	Ref.
Midwives	−5.166^‡^	−1.776^‡^	NA^ǁ^	NA^ǁ^	NA^ǁ^	−1.629^‡^
Nurse assistants	−3.873^‡^	NA^ǁ^	NA^ǁ^	NA^ǁ^	NA^ǁ^	NA^ǁ^
Facility type^†^						
Dispensaries	NA**	Ref.	Ref.	Ref.	NA^††^	NA^††^
Health centers	NA**	1.363^‡^	7.147^§^	8.767^‡^	NA^††^	NA^††^
Hospitals	NA**	0.324^‡^	7.922^¶^	−15.935^‡^	NA^††^	NA^††^

CDI = Cote d’Ivoire; NA = not applicable; Ref. = reference value.

* Ghana and Kenya’s respectful maternal care competency score consisted of one data element. All scores are binary at 0% or 100%.

^†^ The effect of sulfadoxine–pyrimethamine and artemisinin-based combination therapy stock availability was held constant.

^‡^ Not significance.

^§^ *P <*0.001.

ǁ No available data.

^¶^ *P <*0.01.

^#^ *P <*0.05.

** Cameroon observation sample sizes were unequal (health centers, *n =* 328; hospitals, *n =* 11).

^††^ Mali and Niger only had observations at the health center facility level.

## DISCUSSION

Factors contributing to QoC for MiP are highly variable based on country and context, with different facets of context, conditions, and providers driving care competency. Although some relationships shed light on unique dynamics in a particular country, our analysis also highlighted several relationships that transcend borders. Significant positive associations were observed between provider competency and in-stock MiP commodities, as well as key readiness factors, including training and the presence of job aids.

### Malaria in pregnancy commodities and competence.

Commodity availability correlated significantly with MiP prevention competency in three of the six countries. Thus, our analysis dispels the oft-cited attribution of low IPTp uptake to SP stock-outs.^20^ In the six countries included in our analysis, SP availability was generally high (73–97%). That said, the percentage of facilities that had both SP and DOT supplies available on the day of visit ranged from 42% to 86%, with five of the six countries’ availability of both at 75% or less, indicating that providers are often not able to deliver IPTp via direct observation during ANC for more reasons than just a lack of SP. Indeed, several countries’ data showed a significant positive correlation between both SP and DOT supply availability and MiP prevention competency. Notably, the Niger country validation meeting discussion indicated that women often leave ANC with SP to take later because of the low DOT supply availability, although they may be recorded as having received IPTp in the ANC register—a finding reflected in other studies.^21^

Insecticide-treated net availability was generally high and correlated positively with MiP prevention competency in three of the six countries. Although ITN availability correlated negatively with MiP prevention competency in Cameroon, participants in the country validation meeting believe this to be reflective of a data quality challenge. Further assessment is needed to understand more fully the causes of this correlation.

At the time of data collection, the WHO-preferred treatment of uncomplicated malaria in the trimester of pregnancy was quinine + clindamycin for 7 days (or quinine monotherapy if clindamycin is not available), with an ACT or oral artesunate + clindamycin as an alternative if quinine + clindamycin is not available or fails.^20^ All six countries’ policies aligned with WHO recommendations. Availability of quinine was generally poor, with five of the six countries’ availability on the day of visit <75% and three countries’ availability <40%. Ghana was the only country to collect data on clindamycin availability; 47% of facilities visited had clindamycin in stock. Thus, few facilities were able to deliver the WHO-recommended treatment of uncomplicated malaria during the first trimester of pregnancy. Availability of ACTs, the first-line treatment of uncomplicated malaria in the second and third trimesters in all six countries, was >80%, with the exception of Niger at 68% and Cameroon at 55%. However, as the availability of other artemether lumefantrine presentations was not assessed for our analysis, it is possible that other presentations are more available and can be combined to treat MiP when indicated. Availability of injectable artesunate for treatment of severe malaria was variable, ranging from 57% to 94% on the day of visit.

Variable availability of MiP treatment commodities indicates that the WHO-recommended first-line treatment of both uncomplicated and severe malaria in pregnancy is not possible consistently. Unfortunately, most countries do not disaggregate routine malaria treatment data by pregnancy status, including trimester, so there is very little visibility into the classification of MiP cases or the treatment prescribed and administered to pregnant women with malaria. Until this lack of visibility is addressed, it is difficult to ascertain the degree to which MiP is managed appropriately by providers.

### Provider and facility readiness and competency.

Training and availability of job aids correlated significantly with greater competency scores (4–12 percentage points) in several countries across RMC, MiP prevention, and MiP treatment. These findings are not surprising, but highlight the need for continuous support and advancement of health worker knowledge. Although offsite training can be resource intensive, on-the-job training and mentorship can be an effective, targeted way to support health worker competency.^22^ Onsite training also has the potential to reach more providers. During country validation meetings, several countries noted that large-scale malaria and/or MiP training was carried out after these rounds of supervision, likely contributing to increased provider competency moving forward.

Ensuring that MiP guidelines and job aids are available for providers’ easy reference is a lower cost intervention that may yield an important increase in the QoC experienced by pregnant women, and may provide access to appropriate information—a known barrier to IPTp delivery.^21^

Of the three countries for which provider cadre data were available, cadre was correlated only with competency for MiP prevention; Cameroon and Cote d’Ivoire both had significant findings. The country validation meeting elucidated the reasons for these findings, which were driven by provider experience levels in Cameroon and provider access to training in Cote d’Ivoire. Country validation meeting discussions suggested that, in Cameroon, midwives have more years of specialized experience than nurses, which may lead to greater competency scores. Conversely, in Cote d’Ivoire, midwives (more often women than men) have less access to training than nurses, potentially contributing to lower competency (Supplemental Appendix S1). Both countries have already taken, or plan to take, steps to ensure that all cadres of providers have access to the training needed to support greater competency.

Of the three countries for which provider cadre data were available, gender showed a significant correlation with MiP prevention competency only in Cote d’Ivoire, where female health workers scored 5.7 percentage points less than males. This is likely a function of differences between midwife and nurse training access and competency scores; the country validation meeting discussion noted that, in Cote d’Ivoire, midwives are mostly female and nurses included in OTSS+ are mostly male.

Several countries noted that widespread malaria and/or MiP training was carried out after these rounds of supervision, likely contributing to increased provider competency moving forward. In addition, countries took note of individual results for action. For example, Cote d’Ivoire discussed shifting to more on-the-job training to ensure midwives’ skills are reinforced. The Mali team expressed an interest in a future subanalysis comparing public- and private-sector facilities’ MiP service competencies to differentiate and tailor support more effectively. Niger, Mali, and Cameroon all noted that additional training had taken place since the data collection to upgrade MiP service provision skills. In Niger’s case, the country team shared that they had noted that, after the first round of OTSS+, many midwives were not trained; training preference was given to nurses. The team addressed this and since has increased the number of trained midwives. The country teams expressed interest in continuing this type of supervision data analysis to understand and apply the findings more effectively.

### Data limitations and assumptions.

The major limitations of our analysis were that the wording of OTSS+ checklist questions were not standardized and all data elements were not available for all countries. Although Impact Malaria promotes the use of standardized global checklists, OTSS+ checklists are adapted by each country to fit national needs and context. Some countries did not collect gender, cadre, and training data, which limited the scope of our analysis in those countries. In addition, the aggregated data sets were a combination of two OTSS+ checklists: the MiP checklist and the health facility readiness checklist.

Other limitations include 1) supervisor observation may have changed provider behavior by virtue of their presence and 2) stock status reflects the presence of stock on the day of the visit.

## CONCLUSION

Although much of the focus on malaria interventions is on expanding access, health impact will not be achieved without improving simultaneously the QoC when patients reach a facility. In fact, poor-quality care is now a bigger barrier to reducing mortality than insufficient access, whereas high-quality health systems could prevent half of all maternal deaths each year.^23^ The use of comprehensive supervision tools not only contribute to improved clinical practice by serving as a standard rubric for supportive supervision, but also can be an important ongoing data source for countries to assess trends in health worker performance, to identify gaps in service, and to provide actionable information on how to address deficiencies in QoC more effectively. By identifying those aspects associated most strongly with reduced QoC, countries can target resources and tailor interventions more realistically, resulting in the greatest improvements in care. Although commodity availability remains paramount to ensuring appropriate access to interventions and high QoC, identification of additional factors that might otherwise have gone unacknowledged is also critical, such as health worker cadre and gender. More attention may also be focused on relatively low-input interventions, such as ensuring clinical guidelines and job aids are universally available. Improved QoC contributes to optimizing maternal attendance at ANC, thus improving outcomes for both expectant mothers and newborns.

## Supplemental Materials

10.4269/ajtmh.23-0206 Supplemental Materials
