# Supplementary material for: Use of Supervision Data to Improve Quality of Care for Malaria in Pregnancy: Experience in Six African Countries
Source: Am J Trop Med Hyg. 2023 Dec 26;110(3 Suppl):42–9. doi: 10.4269/ajtmh.23-0206 (PMC10919236; doi:10.4269/ajtmh.23-0206)
Supplement: Supplemental Materials [file tpmd230206.SD1.pdf]

Supplemental Information

Use of supervision data to improve quality of care for malaria in pregnancy: experience in six African countries

Running head: supervision data for improved care for malaria in pregnancy

Katherine Wolf[1], Jadmin Mostel[2], Lolade Oseni[1], Patricia Gomez[1], Tabitha Kibuka[2], Courtney Emerson[3], Julie R. Gutman[3], Ashley Malpass[4], Susan Youll[4], Jean Yves Mukamba[5], Eric Tchinda[5], Dorothy Achu[6], Paul Tjek[6], Jean Louis Assa[7], Mamadou Silue[7], Méa Antoine Tanoh[8], Yah Collette Kokrasset[8], Felicia Babanawo[9], Amos Asiedu[9], Mildred Komey[10], Paul Boateng[10], Maureen Mabiria[11], Augustine Ngindu[11], Peter Njiru[12], Ahmeddin Hassan Omar[12], Fatoumata A Sidibe[13], Chebou Diallo[13], Beh Kamate[14], Aissata Kone[14], Sanoussi Elisha[15], Aouna Djibrilla Maiga[15], Alzouma Ibrahim Mayaki[16], Issagana Tidjani Fati[16], Gladys Tetteh[1]

Corresponding author: katherine.wolf@jhpiego.org, PMI Impact Malaria Project, Jhpiego, Baltimore, Maryland, United States of America

Contents

Supplement Figure S1. Overview of OTSS+ data collection and aggregation process. 2
Supplement Table S1. Rounds and sum of observations of OTSS+ data used in the MiP QoC. 3
Supplement Table S2. Outline of variables used in regressions. 4
Supplement Table S3. Components of MiP prevention competency scores... 6
Supplement Table S4. Components of MiP treatment competency scores. 9
Supplement Table S5. Respectful Maternity Care calculations. 11
Supplement Table S6. Classification of health facility types by country. 12
Supplement Table S7. Job aid, training, and DOT supplies indicator language by country....................... 13
Supplementary Text. Country Validation Meeting Findings 15

29 Supplement Figure S1. Overview of OTSS+ data collection and aggregation process.

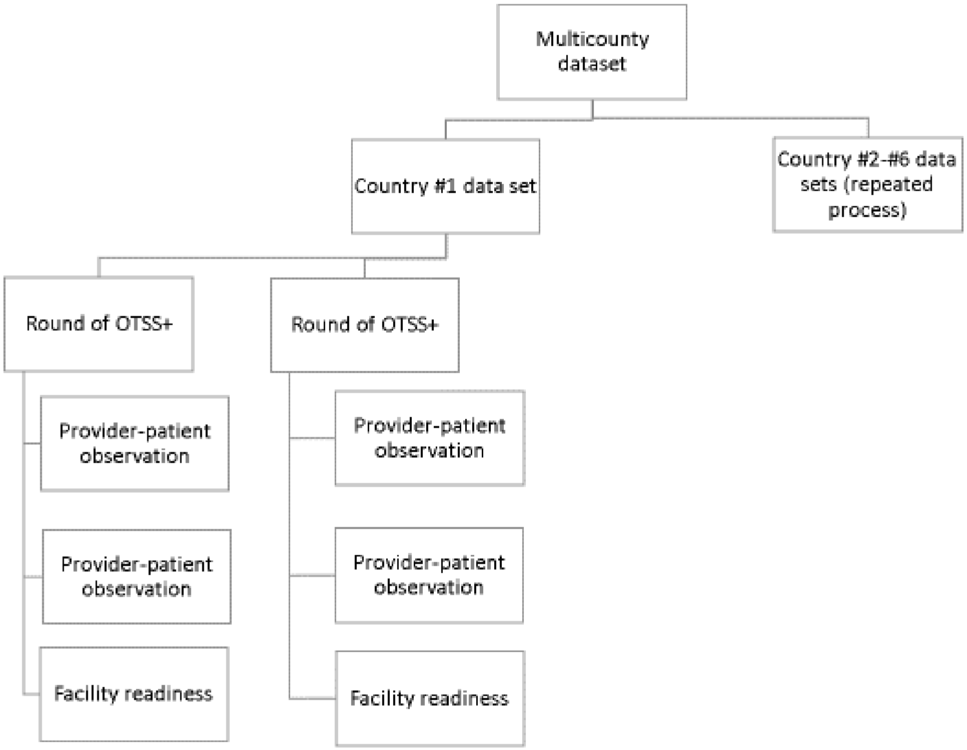

30

Supplement Table S1. Rounds and sum of observations of OTSS+ data used in the MiP QoC.

**Country**	**Rounds of data**	**Total observations**	**Observations by competency score**
Cameroon	Nov–Feb 2020<br>Aug–Sep 2020	339	Prevention: 226<br>Treatment: 94<br>RMC: 333
Cote d'Ivoire	May–Aug 2020<br>Sep–Dec 2020	284	Prevention: 218<br>Treatment: 86<br>RMC: 284
Ghana	Jan–May 2019<br>Sep–Dec 2020	951	Prevention: 944<br>Treatment: N/A<br>RMC: 898
Kenya	Sep–Dec 2019<br>Oct–Dec 2020	82	Prevention: 81<br>Treatment: 81<br>RMC: 81
Mali	Mar–Jun 2020<br>Jul–Oct 2020	595	Prevention: 287<br>Treatment: 283<br>RMC: 595
Niger	Jun–Jul 2020<br>Nov–Dec 2020	193	Prevention: 193<br>Treatment: 193<br>RMC: 193

Supplement Table S2. Outline of variables used in regressions.

Objective	Dependent variables	Explanatory/independent variables	Controlled variables
The effect of availability of essential commodities on overall competency of the provider in offering MiP services	MiP prevention score	SP availability, ITN availability	The other prevention stock variable held constant
	MiP treatment score	Quinine availability, ACT availability, injectable artesunate availability	The other treatment stock variables held constant to examine specific commodities
The effect of directly observed therapy (DOT) supplies (drinking water and cups), training, cadre, facility type, provider gender, and availability of job aids on provider competency in delivery of MiP prevention services	MiP prevention score	Recent training, gender of health worker, availability of DOT supplies (drinking water and cups), availability of job aids, cadre of health worker, health facility type	SP stock availability
The effect of training, cadre, facility type,	MiP treatment score	Recent training, gender of health worker, availability	ACT stock

provider gender, and availability of job aids on provider competency in delivery of MiP treatment services		of job aids, cadre of health worker, health facility type	availability
The effect of training, cadre, facility type, provider gender, availability of MiP guidelines on provider competency in delivery of RMC	RMC Score	Recent training, gender of health worker, availability of job aids, cadre of health worker, health facility type	SP stock availability and ACT stock availability

Supplement Table S3. Components of MiP prevention competency scores.

**Dispensing IPTp**	
1	Did the worker greet the woman at the entrance of the consultation office?
2	Did the worker invite the woman to sit down?
3	Did the worker introduce himself/herself to the woman?
4	Did the worker ask where the woman lived?
5	Did the worker ask the woman's age?
6	Does the worker treat the woman with kindness and respect throughout the visit?
7	Did the worker ask or investigate whether the pregnant woman was at her first ANC? *
8	Did the worker ask about or look for or determine the gestational age (date of last menses, fundal height, sonography) ANC record, pregnancy record)? *
9	Did the worker look for a sign of malaria or ask about a history of fever over the two previous days? And whether the patient presented a fever or not)
10	Did the worker conduct a malaria test (rapid diagnostic tests or microscopy)
11	Did the worker ask or investigate whether the pregnant woman had taken SP in IPTp over the previous four weeks?
12	Did the worker check (mother-child record) whether the woman was on co-trimoxazole for HIV chemoprevention?
13	Has the health worker verified that the pregnant woman is eligible for IPTp during this CPN (gestational age of at least 13 weeks and not having had SP in the previous 4 weeks and

	without co-trimoxazole treatment for HIV prophylaxis)? *
14	Did the health worker made the woman aware of the consequences of malaria on pregnancy outcome?
15	Did the health worker make the woman aware of the benefits of preventing malaria with SP? *
16	Did the health worker educate the woman about the need to take at least three doses of SP before giving birth?
17	Has the health worker informed the woman about the potential side effects of SP? *
18	Did the health worker give three SP tablets to the pregnant woman eligible for IPTp? *
19	Did the health worker ask the woman to drink water to swallow the three SP tablets on the spot? *
20	Did the health worker observe the woman taking the three SP tablets? *
21	Did the health worker ask the woman to return each month for ANC/IPTp until delivery?
22	Has the health worker asked the woman to return to see him/her if there are any serious skin and/or digestive symptoms as a result of taking the SP? *
23	Has the health worker informed the pregnant woman of the date of the next appointment? *
24	Has the health worker recorded the dose of SP in the mother-child record and CPN registry in compliance with the guidelines? *
**LLIN Distribution**	
25	Did the worker explain the benefits of sleeping under an LLIN every night to prevent malaria?
26	Did the health worker give the woman an LLIN if she had not yet received it during this

	pregnancy?
27	Did the worker explain to the woman how to use the LLIN?
28	Did the health worker advise the pregnant woman to sleep under LLINs during pregnancy and after delivery with her newborn baby? *
29	Has the worker recorded the distribution of the LLIN to the woman in the mother-child record and in the appropriate register?
**Dispensing antianemia drugs**	
30	Did the worker give the woman iron tablets after the consultation?
31	Did the worker give the woman 0.4 mg folic acid tablets after the consultation?
32	Did the worker check that the woman understood how to take the iron and folic acid tablets?

Supplement Table S4. Components of MiP treatment competency scores.

**Malaria Diagnosis**	
33	Did the worker explain the result to the woman?
34	Did the health worker ask questions looking for signs of severe malaria (convulsions, agitation, confusion, prostration, etc.)?
35	Did the worker ask if the patient has taken any malaria treatment in the last two weeks?
36	Did the health worker ask about any drugs already taken by the woman?
37	Did the worker take the temperature?
38	Did the worker measure [the patient’s] weight?
39	Did he/she take her blood pressure?
40	Did the worker take a pulse/respiratory rate?
41	Did the worker check for fetal heart sounds (2nd and 3rd trimester of pregnancy)?
42	Did the health worker look for signs of anemia in the woman (pallor of the conjunctivae/palms of hands)?
43	Did the health worker look for signs of other serious illnesses (meningitis, encephalitis, etc.)?
44	Did the worker categorize the type of malaria according to national guidelines?
**Antimalarial Treatment**	
45	**If it is uncomplicated malaria,** does the antimalarial drug used to treat the pregnant woman comply with the guidelines?

46	Did the worker use the correct dosage to treat uncomplicated malaria in pregnant women?
47	Is the dosage and duration of treatment explained to the woman?
48	Did the worker verify that the woman understood the explanations given?
49	Did the worker inform the woman of the potential undesirable effects of the drug used?
50	Did the worker invite the woman to come back if she has complications?
51	Did the worker give the woman and her family appropriate advice on malaria (causes, risks, prevention)?
52	Did the worker give the woman a follow-up appointment?
53	Did the worker enter information on the treatment into the register?
54	**If it is severe malaria,** did the worker administer any pre-transfer treatment?
55	Does the compound used for pre-transfer treatment of severe malaria in pregnant women comply with the guidelines?
56	Did the worker record all the information collected from the woman in his register?
57	Has the woman and/or her family received appropriate malaria counselling?

Supplement Table S5. Respectful Maternity Care calculations.

**Country**	**RMC score calculation:** ***Average of the following data elements***
Cameroon	● Did the provider greet the woman?<br>● Does the provider treat the woman with kindness and respect?<br>● Was the patient and family welcomed/invited to sit down?<br>●Did the health worker encourage the client to ask questions?
Cote d’Ivoire	● Did the provider greet the woman at the entrance of the counselling office?<br>● Did the provider invite the woman to sit down?<br>● Did the provider introduce themselves to the woman?<br>● Does the provider treat the woman with kindness and respect?<br>● Did the provider offer the woman the opportunity to ask questions?
Ghana	●Was the pregnant woman welcomed?
Kenya	●Was the client encouraged to ask questions and were questions answered?
Mali	● Was the woman welcomed with respect and kindness?<br>● Did the provider ask if the pregnant woman had any questions?
Niger	● Did the provider greet the woman at the entrance of the counselling office?<br>● Did the provider invite the woman to sit down?<br>● Did the provider introduce themselves to the woman?<br>● Did the provider treat the woman with kindness and respect?<br>● Did the provider offer the woman the opportunity to ask questions?

Supplement Table S6. Classification of health facility types by country.

	**Dispensary**	**Health Center**	**Hospital**
**Cameroon**	N/A	*Centres de sante integres, centres medicaux d'arrondissement, clinique privee*	*Hôpitaux de district, hopitaux regionaux et assimilés*
**Cote d'Ivoire**	Maternité rurale	*Centre de santé rurale, centre de sante urbain, centre de sante urbain spécialisé*	*Hospital general, centre hospitalier regional*
**Ghana**	CHPS	Clinics, health centers	Hospitals, medical centres, polyclinics
**Kenya**	Dispensaries	Health centers	Sub-county hospitals, hospitals
**Mali**	N/A	*Centre de santé communautaire, centre de sante de référence*	N/A
**Niger**	N/A	*Centre de santé intégré*	N/A

Supplement Table S7. Job aid, training, and DOT supplies indicator language by country.

	Job Aid Availability	Training data	DOT supplies
Cameroon	Are the malaria and pregnancy guide or tools and/or support on ANC available in the facility/ Are the ANC guidelines available today/ Are the ANC guidelines, including the long-lasting insecticidal nets (LLINs), available in the facility on the day of supervision/ Is the guide for the management of malaria in pregnant women available today in the health facility	Have you received training on the national malaria control program's guidelines	Is drinking water available to administer SP/ Drinking water at the ANC site/Are the cups for administering SP during the DOT available/ Disposable cups for the DOT strategy
CDI	Are the malaria and pregnancy guide or ANC tools and/or support available/Are the ANC guidelines available	Have you received training on the national malaria control program's guidelines	Drinking water at the ANC site/Disposable cups for SP DOT strategy during ANC

Ghana	MiP Guidelines Available	Formal training MiP past 6 months/On-the-job training MiP Past 6 months	Is clean water available for directly observed therapy (DOT) of SP at the antenatal care unit
Kenya	Prevention of malaria in pregnancy poster/job aid	N/A	Cups or other drinking devices/Available clean drinking water
Mali	Availability of memory aids on the MiP in the maternity ward	The supervised health worker has received training in MiP within the past 2 years	Availability of potable water and clean cups in the ANC room for supervised SP intake in the ANC room
Niger	Are the malaria and pregnancy guide or tools/support on ANC available/Are the National Malaria Management Guidelines available	N/A	Disposable Glasses for SP DOT Strategy/Drinking water at the ANC site

Supplementary Text. Country Validation Meeting Findings

Country data validation meetings were held after the completion of data analysis and yielded qualitative information about data collection, the health system, and the supervision process to contextualize country-specific findings of the data analysis. Country-specific results from the validation meetings are as follows:

Cameroon

The discussion focused on the findings around MiP prevention competency and ITN availability, and health worker cadre. The negative correlation between ITN availability and MiP prevention competency was attributed to data quality challenges. The team found the correlations between competency and health worker cadre unsurprising. In Cameroon, nurse assistants often have many more years of experience than nurses. Midwives benefit from more in-depth, pregnancy-focused training. The Cameroon team confirmed that more nurses have been trained on MiP and case management since the supervision visits included in the analysis occurred.

Cote d'Ivoire

The discussion with the Cote d'Ivoire team also focused on understanding and responding to difference in competency between cadres and genders; however, in Cote d'Ivoire, nurses and male health workers were observed to have higher competencies than midwives and female health workers. The team confirmed that most nurses are male and most midwives are female. They also noted that nurses tend to work in more rural, smaller health facilities while midwives tend to work in larger, more urban facilities that have a larger staff. When trainings are held, each facility is offered a small number of seats in the training. The result is that all providers in smaller facilities are trained, while only some of the providers in larger facilities are trained. The team noted that a shift to more on-the-job training could address this issue by reaching a larger number of providers.

Ghana

The Ghana dataset contains the largest number of observations compared to the other countries in the analysis. As such, the results from Ghana may be the most representative, generalizable, and robust. These results may be most likely to illuminate relationships between the outcome variables and independent variables, should these relationships exist. SP availability, ITN availability, and DOT supplies have the largest relationship with competency, followed by training. Therefore, investments in these areas may have the largest impact on competency.

Kenya

Kenya's OTSS+ process is considered mentorship, as the national supportive supervision process does not include observation of competency but primarily assesses facility readiness and health worker knowledge of the national malaria guidelines. Because the sample size was small, non-significant relationships **were** identified; however, several regressions seemed indicative of trends worth noting. MiP prevention competency scores were 5.5 percentage points higher when job aids were available. Conversely, the presence of quinine correlated negatively (though non-significantly) with MiP treatment competency, with a 16.6 percentage point reduction in competency score. The country team noted that quinine stock levels had been low, so providers were likely using ACTs, which may lead to this negative relationship. (According to WHO treatment guidelines, this practice is acceptable in the absence of quinine.)

Mali

Mali noted several commodity-related challenges that may have contributed to the lack of significant positive correlation between stock availability and competence. In some cases, pregnant women may be charged for SP, as also happens for ACTs. In addition, private sector facilities (which were included in the OTSS+ supervision) often experience stock-outs of malaria commodities. The Mali team expressed an interest in a future sub-analysis comparing public and private sector facilities' MiP service competencies to better differentiate and tailor support. The Mali team noted that the data analyzed were collected

before widespread trainings were carried out. The country team noted that previously, providers were more comfortable using quinine for treatment of severe MiP.

Niger

The country team noted the negative but non-significant correlation between having SP stock available and MiP prevention competency. The team noted that DOT supplies in Niger remain a challenge, with observed DOT supply availability at 43%. When drinking water is not available, pregnant women are given SP and encouraged to take it at home. While IPTp may be recorded as administered, it’s difficult to know whether the tablets were ever taken. There have been efforts to encourage women to bring drinking water to ANC to facilitate taking SP at the facility. In addition, efforts have been made to reorganize patient flow through the facility so a staff member can confirm that the SP was taken as the woman exits the facility.

The country team shared that they had noted after the first round of OTSS+ many midwives were not trained, as training preference was given to nurses. The team addressed this and since has increased the number of trained midwives. The team noted an improvement in competency following the increase in trained providers.
